# Who moved my eggs? An experimental test of the egg arrangement hypothesis for the rejection of brood parasitic eggs

**DOI:** 10.1007/s10071-014-0800-x

**Published:** 2014-09-07

**Authors:** Daniel Hanley, Peter Samaš, Mark E. Hauber, Tomáš Grim

**Affiliations:** 1 Department of Zoology and Laboratory of Ornithology, Palacký University, 17. listopadu 50, 77146 Olomouc, Czech Republic; 2 Department of Psychology, Hunter College and the Graduate Center, City University of New York, 695 Park Avenue, New York, NY 10065 USA

**Keywords:** Blackbird, Brood parasitism, Egg arrangement, Image processing, Recognition

## Abstract

Avian brood parasitism is an exceptional reproductive strategy whereby parasites reduce their own costs associated with parental care and impose them on the host parents. Consequently, host species have evolved multiple defensive mechanisms to combat parasitism. The vast majority of research attention to date has examined host defenses to recognize and reject parasitic eggs. The recently proposed “egg arrangement hypothesis” suggests that hosts may not focus solely on individual eggs’ features, but instead the overall arrangement of the clutch may also provide a cue that parasitism has occurred. Correlative data revealed that host females maintaining a consistent egg arrangement across the incubation period were more likely to reject foreign egg models than females that did not keep a consistent egg arrangement. Here, we provide the first experimental test of this hypothesis in the European blackbird (*Turdus merula*). We experimentally parasitized nests such that the egg arrangement was either disrupted or not disrupted. We found no evidence that altered egg arrangement was used as a cue for egg rejection by host females. Therefore, we suggest that females that keep consistent egg arrangement are more likely to eject foreign eggs for other correlated reasons. Thus, egg arrangement does not serve as an independent cue to trigger egg rejection responses to parasitism in this host species.

**Electronic supplementary material:**

The online version of this article (doi:10.1007/s10071-014-0800-x) contains supplementary material, which is available to authorized users.

## Introduction

Brood parasites lay their eggs within another female’s nest, and force these host birds to become foster parents for their offspring (Davies 2000). By doing so, brood parasites shirk all responsibility and costs associated with caring for their offspring and impose them on host parents (Hauber and Montenegro 2002). Consequently, hosts have evolved sophisticated and multiple defensive mechanisms to respond to the risks and costs of brood parasitism (Rothstein 1975; Davies and Brooke 1989; Grim et al. 2011). The host faces three types of challenges to combat parasitism in the nest: (1) the sensory task of discriminating between own versus foreign eggs within the clutch, (2) the cognitive task of recognizing the parasitic egg(s), and (3) the motor task of rejecting parasitism, through either deserting a parasitized clutch or ejecting the foreign egg(s) by grasping, puncturing, or burying (Lyon 2003; Stokke et al. 2005; Soler et al. 2012).

Birds may discriminate parasitic eggs by comparing the eggs found within their clutch to a learned or inherited archetype (template matching) and/or identifying the egg(s) with an outlying phenotype (discordancy; Moskát et al. 2014). Although a variety of factors are known to influence egg recognition, much variation in host responses to reject or accept foreign eggs remains unexplained (Moksnes et al. 2013). Recently, Polačiková et al. (2013) suggested that host parents may use indirect information gained from examining disruptions to the arrangement of their eggs as a cue that something has occurred to their clutch during their absence from the nest. Specifically, the parasite female may move the host eggs because she lands on the nest, which can move the nest and the clutch, especially in cases when the nest is built on unstable vegetation (e.g., reeds or bushes as opposed to ground or robust tree branches) and/or when the parasite is larger or heavier than the nest owner (e.g., common cuckoo *Cuculus canorus* vs. *Acrocephalus* warblers, Wyllie 1981). Additionally, some parasites (e.g., common cuckoo) typically remove at least one host egg and add a parasitic egg (Moksnes et al. 2000), which inevitably alters the egg arrangement.

Interestingly, Polačiková et al. (2013) suggested that female European blackbirds (*Turdus merula*; hereafter: blackbirds) and song thrush (*T. philomelos*) in New Zealand that keep their arrangement relatively consistent tend to reject foreign eggs, while those that often change the arrangement tend to accept foreign eggs. However, because these data were correlative, it is also possible that egg arrangement was influenced by extrinsic physical factors (e.g., branch vibrations caused by wind). In addition, a female’s ability to neatly arrange a clutch may be a component of the host’s phenotype related to individual behavioral suites (Sih et al. 2004; Trnka and Grim 2014), together with broodiness or attentiveness, which may make a female more likely to detect and respond to the parasitic egg using an egg-based recognition mechanism (e.g., template matching or discordance). Alternatively, clutch consistency may correlate positively with the accuracy of other female cognitive processes, so that females with superior egg recognition abilities also keep more consistent clutches.

Here, we performed an experiment examining whether disruptions to egg arrangement influenced the rejection rate and latency to rejection, in a European population of blackbirds. At clutch completion, we sequentially assigned blackbird nests to a control or one of two treatment groups. For both treatment groups, we added a non-mimetic model egg to clutches at the perimeter of the cup without reducing the original host brood size, because replacement with or addition of parasitic eggs has no effect on host egg rejection responses in this species (Davies and Brooke 1989; Grim et al. 2011). We also included control nests where no experimental parasitism occurred. For our treatments, we either introduced the foreign egg model without disrupting the arrangement of the clutch or we disrupted the arrangement after artificial parasitism. The control nests and these treatments should provide three distinct classes of clutch disruption: no disruption, subtle disruption, and large disruption to egg arrangement. We predict that, if blackbirds use egg arrangement as a cue that a parasitism event has occurred, greater disruption of egg arrangement should result in a greater proportion of rejected eggs and a shorter latency to rejection.

## Methods

### Study area and experimental procedures

We conducted the study in the city of Olomouc, Czech Republic (49°35′38″N, 17°15′3″E) April–June 2013. We focused on blackbirds, because correlative work (Polačiková et al. 2013) suggested that the consistency of egg arrangement could be an important cue for egg rejection decisions in this species. We searched for nests (*N* = 222) and focused on those that reached clutch completion without failure and were not used in other experiments (yielding *N* = 79 nests for this study). Whenever possible, we recorded laying dates directly (from daily nest checks, *N* = 24) or estimated them from the clutch size, clutch completion, and hatching dates (*N* = 55) assuming one egg laid daily and a 13 day incubation period (our own unpublished data from the study population). Neither female response nor latency to rejection was influenced by the estimation of laying date (Electronic Supplementary Material 1). The nest age when manipulation occurred (hereafter nest age; days ± SE; 4.65 ± 0.42 days) was determined relative to the clutch completion date (day 0). Clutch size was either four or five eggs, which is typical in this population (Samaš et al. 2013).

We used the same type of plain light blue model egg that was used by Polačiková et al. (2013). The size (mean ± SD = 22.40 ± 0.34 mm × 16.89 ± 0.29 mm, *N* = 32), mass and spectral reflectance of these models provide a close match to cuckoo eggs naturally found in common redstart *Phoenicurus phoenicurus* nests (further details in Samaš et al. 2011). The model egg was introduced into blackbird nests upon clutch completion under three experimental treatments: control (*N* = 19, hereafter control), no rearrangement (*N* = 30, hereafter constant), and shuffled egg arrangement (*N* = 30, hereafter rearranged). At control nests, the researcher (DH) held his hand over the nest cup without touching the eggs for 10 s. At constant nests, a parasitic egg was added to the edge of the cup in the nest after the researcher (DH) held his hand over the nest, so that the total time spent above the clutch was 10 s. At rearranged nests, the model was added to the nest after the researcher (DH) carefully shuffled the eggs by hand using a figure eight pattern for 10 s. No eggs were damaged by these manipulations. To avoid influencing the natural arrangement, the eggs were not handled, numbered, or measured prior to experimentation, and the nests were monitored daily with a telescopic mirror to avoid direct contact with either the nest or clutch. We photographed a subset of nests (8 of 19 control, 17 of 30 constant, and 25 of 30 rearranged clutches) both before and after experimental manipulation with an Olympus E-PL1 camera, using automatic settings and storing images in JPEG format.

After manipulation, all nests were monitored until egg ejection or for six days if the foreign egg was accepted (six days is a standard period in egg discrimination studies: Davies and Brooke 1989; Grim et al. 2011). Control nests were followed for six days (Samas et al. 2014). In this study, all ejections were of the foreign egg model and no ejection errors occurred (i.e., no blackbirds ejected their own egg instead of the foreign egg model). In addition, we detected no instances of natural parasitism, either conspecific or interspecific, in any of these nests.

### Image analysis to quantify egg arrangement

To validate that our experimental manipulation affected egg arrangement, we quantified the egg arrangement from our photographic data following previous protocols (Polačiková et al. 2013) as well as used a novel technique to compare pattern variation (Taylor et al. 2013). For each egg in each photograph, we quantified four distinct features describing egg arrangement using a custom ImageJ (Schneider et al. 2012) macro (Electronic Supplementary Material 2): blunt pole distance, blunt pole angle, blunt pole orientation, and adjacent angles (see Fig. 1 in Polačiková et al. 2013). Blunt pole distance is the distance between the nest center and the egg’s blunt pole, blunt pole angle is the angle created by the positive *x* axis and the vector connecting the blunt pole and nest center, blunt pole orientation is the angle created by the positive *x* axis and each egg’s long axis, and the adjacent angles are the angles created between the long axes of adjacent pairs of eggs and measured between the long axis of egg *N* to egg *N* + 1 in a clockwise direction. To determine how much the egg arrangement was changed by our experimental manipulations, we used the before and after manipulation photographs to calculate the standard deviations for each of these metrics, and we used these values for further analyses. In addition, we quantified the arrangement of the entire clutch following a new method for image processing called the distance transformation (Taylor et al. 2013) using custom scripts in ImageJ and ImageMagick (Electronic Supplementary Material 3; to install ImageMagick visit http://imagemagick.org). This method assesses the similarity between two binary images, while accounting for subtle differences in image alignment and size. This resulted in values (hereafter dissimilarity) that represent the difference (in proportion) between the before and after image.

**Fig. 1:**
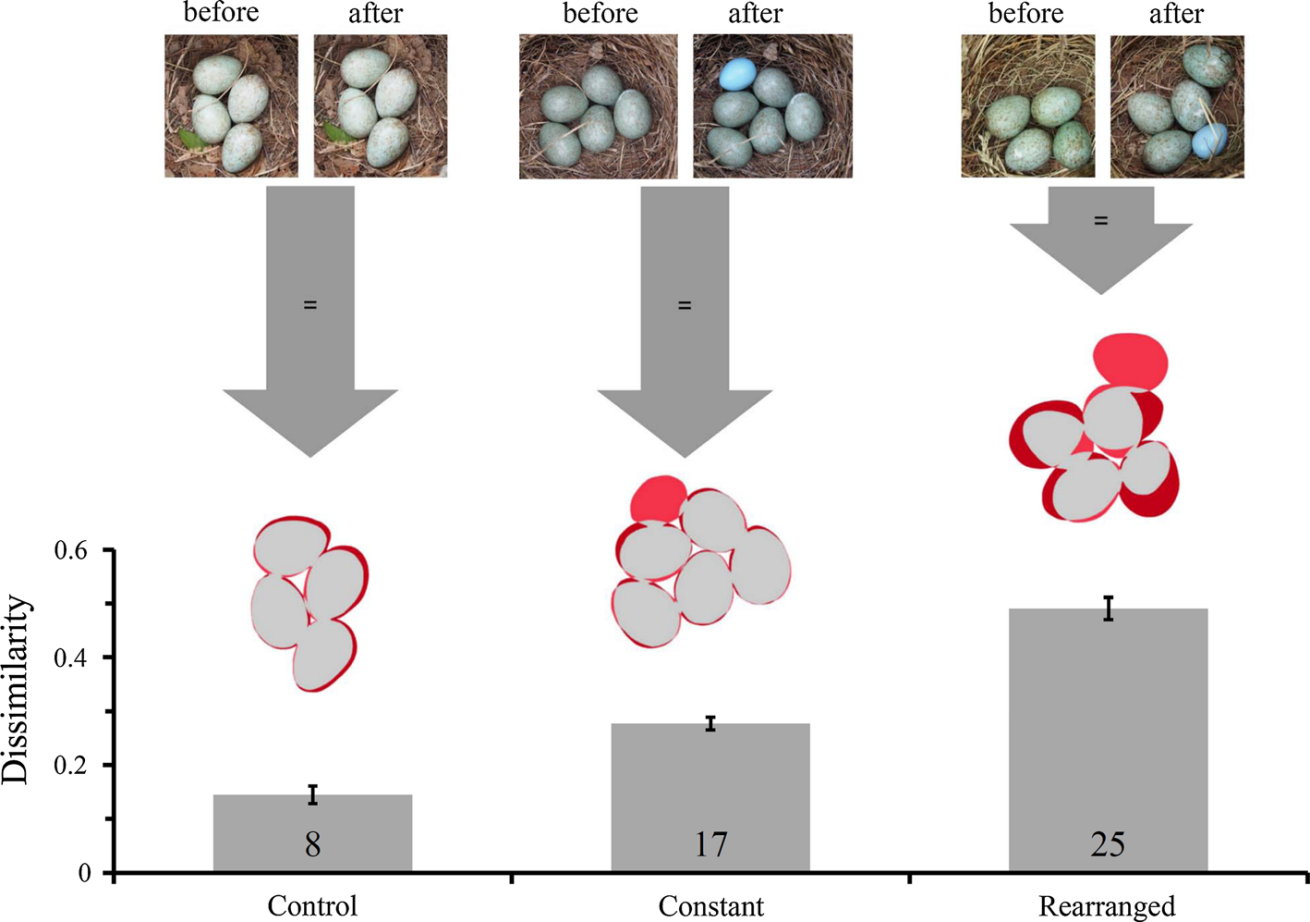
Visual and quantitative illustration of the dissimilarity score of clutches before and after the experimental manipulation of clutches (*photographic insets*) that were not disrupted and where no foreign egg model was added (control), clutches that were not disrupted and a parasitic egg was added to the nest (constant), and clutches where egg arrangement was disrupted and a parasitic egg was added (rearranged). Here, dissimilarity is calculated as the proportion of mismatch between the original clutch and the post-manipulation photograph. We depict areas of similarity between pairs of photographs in *gray* (*light gray* in print) and areas of dissimilarity between the photographs in shades of *red* (shades of* dark gray* in print) on images of clutches that are representative nests of each group. The *bars* represent the mean ± SD. *Numbers inside the bars* represent sample sizes (color figure online)

### Data analysis

Desertion was unrelated to manipulation (see Results) therefore we used only non-deserted nests in our analyses. We considered parental response a binary response (either egg ejection or acceptance); however, including desertion as a potential response (cf. Hauber et al. 2014; Samas et al. 2014) did not alter our conclusions (results not shown). To determine whether our experimental treatments resulted in different host responses to artificial parasitism, we used a generalized linear model with a binomial error distribution and logit link function controlling for the effect of potential predictors, including nest age (continuous), first egg laying date (continuous), and clutch size (categorical) that could influence a parent’s ability or motivation to respond to our experimental treatments. To predict latency to ejection (number of days as a count response), we performed similarly constructed models using the same model selection procedures and covariates; however, for these analyses we used generalized linear mixed models with a negative binomial error distribution and log link. We used a backward elimination procedure (Grafen and Hails 2002), where we removed the least significant predictor from each model, until we had a reduced model with significant predictors and the predictor of main interest, treatment. This treatment predictor was always kept in the model regardless of its significance. We present both the full and reduced models. Generalized linear models were conducted with the “glm” function in the “stats” package for models with binary responses and with the “glm.nb” function in the “MASS” package for models with negative binomial responses (Venables and Ripley 2002) using the programming language and software environment, R, version 3.1.0 (R Core Team 2014).

## Results

**Fig. 2:**
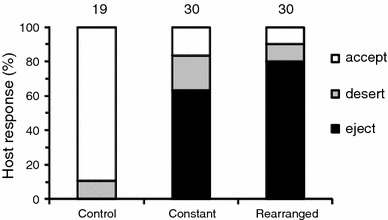
Behavioral responses (nest desertion, and egg acceptance or ejection) of European blackbirds assigned to control, constant, and rearranged treatments. Here, non-deserted control nests are depicted as accepted. Sample sizes are provided above the *bars*

We confirmed that our treatments successfully manipulated all egg arrangement metrics using two separate approaches (for further details see, Electronic Supplementary Material 1; Fig. 1). We found that desertion rates were similar in control (10.5 %, *N* = 19), constant (16.7 %, *N* = 30), and rearranged clutches (10.0 %, *N* = 30). A Fisher’s exact test for count data, with Monte Carlo simulated *P* values (using 100,000 replicates) confirmed that there was no difference between the number of control or treatment nests (constant and rearranged clutches combined) which were deserted (Odds ratio = 0.67, CI_0.95_ = 0.06–3.73, *P* = 1.00; Fig. 2). Therefore, desertion was not a direct response to the introduction of this particular foreign egg model and deserted nests were excluded from further analyses.

Ejection responses from non-deserted nests (Fig. 2) were similar in both the constant (79.2 %, *N* = 24) and rearranged treatment (88.9 %, *N* = 27). Our two experimental treatments had no differential influence on host response (Table 1). Similarly, our two manipulations had no influence on latency (Table 1), which ranged from the same day to 6 days (median = 1 day); however, females with larger clutches ejected eggs significantly faster than females with smaller clutches (Table 1). Tests examining how the exact amount of clutch rearrangement influenced host egg ejection responses and latency to ejection produced very similar results (Electronic Supplementary Material 1).

**Table 1:** Generalized linear model outputs predicting the behavioral response to experimental parasitism (either egg ejection or acceptance) and its latency (for egg ejections only)

	Full model					Final model				
	Estimate	Approximate CI 95 %	*χ* ^2^	*P*	VIF	Estimate	Approximate CI 95 %	*χ* ^2^	*P*	VIF
Response *R* ^2^ = 0.09						*R* ^2^ = 0.03				
(Intercept)	45,439.21	−42,066.60 to 132,945.02		0.31		1.34	0.35–2.32		**0.01**	
Treatment	0.55	−1.20 to 2.29	0.39	0.53	1.21	0.74	−0.81 to 2.30	0.91	0.34	
Nest age	−0.12	−0.34 to 0.10	1.09	0.29	1.17					
Laying date	−0.02	−0.07 to 0.02	1.05	0.31	1.05					
Clutch size	−0.04	−1.84 to 1.77	0.00	0.97	1.34					
Latency to ejection *R* ^2^ = 0.36						*R* ^2^ = 0.29				
(Intercept)	32,550.69	−8,465.56 to 73,566.93		0.12		0.37	−0.24 to 0.98		0.24	
Treatment	0.57	−0.20 to 1.35	2.00	0.16	1.17	0.74	−0.06 to 1.54	3.25	0.07	1.16
Nest age	−0.03	−0.13 to 0.08	0.21	0.65	1.19					
Laying date	−0.02	−0.04 to 0.004	2.40	0.12	1.07					
Clutch size	−1.12	−1.93 to −0.31	7.64	**0.006**	1.31	−1.21	−2.00 to −0.42	9.20	**0.002**	1.16

We show the regression estimates, their approximate 95 % family-wise confidence intervals, significances (bolded if below the significance criterion of 0.05), and their variance inflation factor, for both the full model and reduced model arrived at from a backward elimination process

## Discussion

Most research on brood parasitism has focused on defenses against parasitic eggs (Fig. 1 in Grim 2007) and most of those studies testing the cues triggering host egg discrimination responses focused on phenotypes of individual host and parasite eggs (i.e., their dis/similarity: Bán et al. 2013) or cues external to the host nests (i.e., parasite density: Welbergen and Davies 2012). Here, we provide the first experimental test of the “egg arrangement hypothesis” (Polačiková et al. 2013) to determine whether disruptions to egg arrangement influence rejection rates and latency to rejection in a model species, the blackbird. Despite sample sizes that are comparable or larger than those in experimental manipulations in most egg rejection studies (Grim 2007; own unpublished review of sample sizes in brood parasitism studies), we found no experimental support for the egg arrangement hypothesis. Altering the arrangement of eggs during an artificial parasitism event made no difference in the behavioral responses, or the latency to rejection, displayed by the host in response to the foreign egg.

Previous research found that ejecters maintained consistent distances between the nest center and the blunt poles of their eggs, and that the variation (measured as SD) of some adjacent angles were lower in ejecters than acceptors (Polačiková et al. 2013). We found no evidence to suggest that disruptions to these traits influenced host parents’ likelihood to eject or the latency to ejection, which suggests that in Polačiková et al. (2013) ejecter females that maintained consistent arrangement of blunt pole distances and adjacent angles were not using these as independent cues. Instead, these traits were most likely related to other female characteristics. Although the original study (Polačiková et al. 2013) was conducted in New Zealand in the absence of common cuckoo parasitism pressure and our study was conducted in the blackbird’s native European range, it is likely that blackbirds should have responded similarly in both locations. Blackbirds neither were historically nor are currently regularly parasitized by the cuckoo within their native range (Moskát et al. 2003; Grim et al. 2011) and are not parasitized by any interspecific parasites in their New Zealand ranges (Samas et al. 2014). The native and introduced populations do not differ in any of their relevant anti-parasitic adaptations to non-mimetic eggs: egg ejection rate, nest desertion rates, latency to egg ejection, or repeatability of egg ejection (Grim et al. 2011, 2014; Samaš et al. 2011; Samas et al. 2014). Recent evidence suggests that blackbirds have evolved egg rejection behaviors in response to conspecific parasitism (Samas et al. 2014), which is known to occur in both their native and introduced ranges. Thus, both European and New Zealand populations are equally suitable for tests of the egg arrangement hypothesis with no difference being predicted for populations sympatric or allopatric with any interspecific brood parasites (see also Grim et al. 2011).

Furthermore, it is possible that the consistency of egg arrangement previously reported (Polačiková et al. 2013) was maintained by nest characteristics (e.g., stability against wind, etc.) that correlated with female ejection ability. However, this is an unlikely explanation because extrinsic disruptions inevitably experienced by blackbirds tested in Polačiková et al. (2013) would not disrupt blunt pole distance or adjacent angle without also influencing the other metrics of arrangement. In addition, our lack of experimental support may be because we examined females that kept either consistent or inconsistent clutches (where egg arrangement may be important and relatively less important, respectively). Our treatments were randomized and therefore if arrangement was used in this population, some effect should have been detectable, unless all birds in our population kept inconsistent clutches; although, it is possible that, despite large sample sizes, we were only able to detect large effects, particularly for our examination of female response (i.e., ejection vs. acceptance). Instead, our results most likely suggest that egg arrangement does not affect blackbird responses to foreign eggs, and that a female’s ability to maintain a consistent egg arrangement may indirectly relate to her recognition capabilities.

Although we do not provide experimental support of the egg arrangement hypothesis, this does not exclude that it may be important to other populations or host species. Future tests of this hypothesis would also benefit from examining two assumptions of the egg arrangement hypothesis. For egg arrangement to serve as an effective cue of a parasitism event, the deposition of a parasitic egg must disrupt the arrangement of the host’s clutch more than natural events during the absence of the incubating bird or the disruption created by the bird leaving the nest. In our experience, birds leave their nests rapidly when flushed, but during natural recesses they leave their nests carefully (J. Weiszensteinová and T. Grim, unpublished data). Our results show that simply adding the foreign egg model without intentionally disrupting the arrangement of the clutch (i.e., the “constant” treatment) did change to some extent the original egg arrangement, suggesting greater disruptions during natural parasitism events. However, although our disruptions to egg arrangement were random, disruptions caused by brood parasites may change egg arrangement in a non-random way. This possibility will need to be investigated within a natural context in the future. Currently, there are very few analyses on how and when parasitic eggs are added to clutches (Wyllie 1981; Moksnes et al. 2000; Lyon 2003) and no analyses to assess how egg arrangement changes after natural brood parasitism events. In fact, only a few studies have examined in detail both parasite and host behavior during real parasitism events (e.g., Moksnes et al. 2000; Tewksbury et al. 2002; Ellison and Sealy 2007; Gloag et al. 2013; Soler et al. 2014).

The egg arrangement hypothesis also assumes that birds make visual or tactile evaluations of the arrangement of the clutch prior to leaving and upon returning to their nests. Birds are known to inspect their clutches both when they return to their nests (Honza et al. 2004; Antonov et al. 2009; Moskát et al. 2014) and throughout their incubation bouts (Honza et al. 2004; Požgayová et al. 2011). It is possible that the arrangement of the clutch is evaluated during these inspections, but it is also possible that birds are assessing clutch size. Previous studies on the great reed warblers (*Acrocephalus arundinaceus*) have found that post-manipulation desertion was higher when the initial clutch size was low (Moskát et al. 2011) and that hosts rejected fewer parasitic eggs when they had larger clutches due to increased risk of errors (Moskát and Hauber 2007). In contrast with these results, we found that desertion was not a response to parasitism (cf. Moskát et al. 2003) and that females with larger clutches ejected no more or fewer foreign eggs than those with smaller clutches; however, females with larger clutches ejected foreign eggs more rapidly.

The egg arrangement hypothesis may be well suited for future comparative investigations. Using the arrangement of clutch as a parasitism cue requires that the species is parasitized, the arrangement of the clutch is generally consistent (except after egg deposition), the clutch is sufficiently large to provide useful arrangement cues, and the degree of mimicry is high. Ideally, future researchers will examine video and photographic data on parasitism events, egg arrangements, and host responses, across a range of hosts that differ in these characteristics.

In conclusion, despite the potential adaptive benefit of using egg arrangement as a cue of parasitism and contrary to previous correlative results, we found no support for the egg arrangement hypothesis. Our experimental results suggest that the previously reported correlative findings (Polačiková et al. 2013) illustrate that female recognition abilities are simply correlated with her ability to maintain egg arrangement, but that arrangement is not a cue *per se* in European blackbirds.

## Electronic supplementary material

Below is the link to the electronic supplementary material.

Supplementary Methods and Results including detailed information on how egg arrangement was quantified from nest photographs. In addition, we provide validation that the experimental manipulation successfully altered egg arrangement, and additional tests that examine parental response to the absolute amount of clutch disruption. (DOC 183 kb)

Custom ImageJ macro to calculate egg arrangement. To use this function in ImageJ, click Plugins, Macros, and Install and then select this macro. (IJM 2 kb)

ImageMagick codes used to calculate dissimilarity (for details see Methods). To use these codes you must first have ImageMagick installed, and then extract this zipped file. These are bash codes intended to be run on a Linux operating system, but these can be customized for other operating systems. (ZIP 13 kb)
