# Supplementary material for: Who moved my eggs? An experimental test of the egg arrangement hypothesis for the rejection of brood parasitic eggs
Source: Anim Cogn. 2014 Sep 7;18(1):299–305. doi: 10.1007/s10071-014-0800-x (PMC4674666; doi:10.1007/s10071-014-0800-x)
Supplement: Supplementary file 1 — Supplementary Methods and Results including detailed information on how egg arrangement was quantified from nest photographs. In addition, we provide validation that the experimental manipulation successfully altered egg arrangement, and additional tests that examine parental response to the absolute amount of clutch disruption. (DOC 183 kb) [file 10071_2014_800_MOESM1_ESM.doc]

**Electronic Supplementary Material 1**

Supplementary Methods & Results

*Estimation of nest age*

A Fisher’s exact test for count data, with Monte Carlo simulated *P* values (using 100,000 replicates) found that female response to the model was unrelated to whether laying date exactly known or estimated (*P* = 0.52). A negative binomial generalized linear model found that latency to response did not differ between nests with laying dates known (days ± *SE*; 1.06 ± 0.33 days) vs. estimated (days ± *SE*; 1.33 ± 0.23; *χ^2^*= 0.29, *P* = 0.59). While the candling techniques used in Polačiková et al. (2013) might have provided more precise estimates (Lokemoen and Koford 1996), the disruption of natural arrangement due to handling of eggs would subvert our study design in the constant and rearranged treatments groups.

*Image analysis to quantify clutch arrangement*

Wherever possible (due to nest location), nests were photographed using an Olympus E-PL1 camera, using automatic settings and storing images in JPEG format. Photos were taken from over the nest cup immediately before and after manipulation. Due to inaccessibility we photographed 42% (*N* = 8 of 19) of the control nests, 57% (*N* = 17 of 30) of the constant clutches, and 83% (*N* = 25 of 30) of the rearranged clutches. A coin was used as a size standard and placed on the edge of each nest photographed; this prevented disruption of the clutch, but can introduce some noise into estimates of clutch arrangement when nest cups vary with depth. We then quantified the clutch arrangement following two protocols: the methods used by Polačiková et al. (2013) and a novel technique to compare pattern variation (Taylor et al. 2013). Both methods required pre-processing of nest images. For this step we combined before and after manipulation photographs in a layered TIFF format image for each nest (Figure 1). These separate layers were then size calibrated using ImageJ (Schneider et al. 2012). The nest centers were identified using common landmarks in both images, and the layers were then aligned by translation and rotation. Although some machine vision techniques exist to align images objectively (Tseng et al. 2011), we opted for an alternative and internally consistent technique because these objective techniques require constant landmarks in the comparison photographs. Due to the experimental manipulations landmarks such as features within the nest were not identical in the two subsequent photographs because of the experimental rearrangement. In addition, it is difficult to perfectly standardize the distance between the camera and the focal nests and the viewing angle (note that blackbird nests are typically located in dense bushes or trees). Moreover, this method closely approximates the previous method (Polačiková et al. 2013) and the inconsistencies with respect to scale and distance were compensated for by our second approach (Taylor et al. 2013). However, all differences were examined relative to the control group, which should also be similarly influenced by our subjective assessments and therefore we do not expect that these errors will systematically bias our results in any particular direction.

Using the first approach (Polačiková et al. 2013), we applied a coordinate plane such that the origin was placed upon the nest center in both the before and after nest photograph. Then, we numbered eggs starting directly right (relative to the orientation of the nest on the photograph) of the nest center and proceeding in a counterclockwise direction. These eggs were assigned numbers that were used to identify individual eggs (hereafter, egg ID) in subsequent analyses. However, it is important to note that egg ID does not represent the true identity of eggs but rather identify an egg in a particular location within the clutch, just like in the study of Polačiková et al. (2013).

For each egg we examined four distinct features describing clutch arrangement: blunt pole distance, blunt pole angle, blunt pole orientation, and adjacent angles (see Figure 1 in Polačiková et al. 2013). As stated in our main Methods section, the “blunt pole distance is the distance between the nest center and the egg's blunt pole, blunt pole angle is the angle created by the positive x axis and the vector connecting the blunt pole and nest center, blunt pole orientation is the angle created by the positive x axis and each egg's long axis, and the adjacent angles are the angles created between the long axes of adjacent pairs of eggs and measured between the long axis of egg N to egg N+1 in a clockwise direction”. These components were calculated using a custom ImageJ macro (Electronic Supplemental Material 2). Because subjectivity in nest alignment may result in misclassification of egg ID (e.g., egg ID #1 from the before photo may appear in quadrant four in the after photograph), even in control nests where eggs did not move, we used an algorithm to re-assign egg ID such that the absolute differences among the eggs between the photos being compared were minimized. Therefore, in our final analysis egg ID was assigned relative to the blunt pole distance, blunt pole angles and orientation, as well as the adjacent angles. Even if the eggs were not the same physical eggs, egg ID is based on the relative position of eggs within the clutch, which is less prone to error than the arbitrary assignment of a coordinate plane (Polačiková et al. 2013).

For these metrics, the standard deviations (*SD*) of the before and after images were used in further analyses; however, due to the circular nature of these data when a particular egg ID fell on either side of the first-fourth quadrant boundary in subsequent photographs we corrected the *SD* rather than merely egg ID. Our goal was to classify the deviation in degrees of egg rotation, therefore we corrected these cases to reflect that. For example, after such a correction eggs with before and after measurements of 1 and 6 degrees and 359 and 4 degrees would both reflect movement of 5 degrees. Without such a correction their *SD* would differ considerably (3.5 and 251.0, respectively). Therefore, we corrected these values so that the *SD* reflected the true deviation between the two measurements. To do this, we added the difference between 360 and the angle in quadrant four (always assigning 360 degrees equivalent to 0 degrees), which set all *SD* values on the same scale. This issue influenced only blunt pole angle and orientation, which were defined by a vector drawn from the origin. Unlike the metrics themselves their *SD* has no directionality. In contrast blunt pole (hereafter, BP) distance was measured in distance (cm) and adjacent angle referenced angles between eggs not the coordinate grid, and therefore these metrics were not influenced by this issue.

We also quantified the arrangement of the entire clutch using a new method for image processing (Taylor et al. 2013) that uses a distance transformation to assess the similarity between two binary images. Instead of using image matching algorithms that are based on feature extraction (e.g., *Fourier transformations*: Zhang and Lu 2002), which have been successfully employed on eggshell image analysis (Stoddard and Stevens 2010), this alternative method follows a template matching approach (Zitová and Flusser 2003) that should not to be confused with egg recognition templates. In contrast to previous methods applied to study host-parasite interactions, we were not assessing the individual features of eggs but instead in the physical location and orientations of the eggs within the clutch, which make the distance transformation an ideal approach. In contrast to alternative methods the distance transform is more sensitive to differences in specific features, rather than overall attributes of patterns between the images (Taylor et al. 2013).

One benefit of this method is that it accounts for subtle differences in image alignment and size. The distance transform method accounted for differences between images in translation (shifts along either axis) and/or scale in a single dimension (stretching along a single axis). Instead, here we created custom scripts to employ the distance transformation but our codes account for photographic inconsistency in translation and scale (both x and y axes). For this, we isolated the silhouettes of the entire clutch from size calibrated images using custom scripts in ImageJ, and then analyzed the differences in custom scripts written for ImageMagick (Electronic Supplemental Material 3; to install ImageMagick visit http://www.imagemagick.org). Specifically, when comparing two images we incrementally varied the relative scale of the “after” image (up to ± 15%) and shifted the image along the x and y axis (up to ± 30% of the pixels), each time saving the transformed “after” image. On each iteration, we compared these transformed “after” images to the “before” image, and saved the transformation that resulted in the smallest absolute difference. For these comparisons, we applied a minimum 5% multi-dimensional spherical distance between “before” and “after” comparison images to account for minor differences introduced to the photograph. In other words, the pixels attributed to eggs in the before image will be considered the same as the pixels in the after image if the difference between the silhouetted eggs in both images is less than five percent. The transformed “after” image with the smallest absolute error (fewest number of mismatched pixels) from the “before” image was used in subsequent processes, and then these steps were repeated to result in an optimal match. After finding the optimal alignment between the two images, the absolute error was divided by to the number black pixels (eggs) in the “before” image. These values (hereafter, dissimilarity) produced by this method represents the difference (in percentage) between the before and after image. While our images differed slightly in rotation, these differences were minor (for examples see, Figure 1) and all groups including the control group were subject to this source of error; therefore, this should not consistently bias our results.

*Data analysis*

All analyses were conducted in R 3.1.0 (R Core Team 2014). For all analyses, we ran separate models for each of our metrics of disruption because they were inter-correlated (Table 1). To determine if our treatments successfully disrupted clutch arrangement, we applied linear mixed models to examine if the *SD* of BP distance, BP angle, BP orientation, or adjacent angles within each nest varied by treatment, controlling for the random effect of nest. We assessed the normality of all response variables using the Shapiro-Wilk test of normality (Shapiro and Wilk 1965), and achieved normality by Box-Cox transformations (Box and Cox 1964) if necessary. Each nest only had a single dissimilarity score; therefore, we used similarly constructed linear models to examine if our treatments sufficiently disrupted dissimilarity. For these models significance between all groups was assessed using a Tukey honestly significant difference test. These test showed that our treatments successfully affected the *SD* of BP distance, orientation, angles, and adjacent angles (all *χ^2^* > 46 all *P* < 0.0001; data not shown) as well as the levels of dissimilarity (*F_2,54_* = 96.5, *R^2^* = 0.78, *P* < 0.0001; Figure in Main text 1).

In addition to examining if the parental response to eggs was predicted by the treatment, we examined if parental response could be predicted by the absolute amount of rearrangement based on before and after photographs, as well as other potential predictors including nest age (continuous), first egg laying date (continuous), and clutch size (categorical) that could influence a parent's ability or motivation to respond to our experimental treatment. For models predicting parental response (acceptance or ejection) we used a generalized linear mixed model with a binomial error distribution with a logit link function, coding nest ID as the random effect. Similar to our generalized linear mixed models presented in the main text, we used a backward selection procedure to produce reduced models, and present these results along with the full model results. These generalized linear mixed models were conducted using the 'glmmADMB' package (Skaug et al. 2011; Fournier et al. 2012), and produced satisfactory model diagnostics. Because every nest had only one value of dissimilarity, models that included dissimilarity were analyzed with similarly constructed generalized linear models without nest ID as random effect and followed a similar backward elimination procedure. Similar to our analyses focusing on absolute clutch disruption (main text), neither the latency to ejection nor parental response (ejection or acceptance) was predicted by the absolute amount of clutch rearrangement for any metric of clutch arrangement (Table 3).

REFERENCE LIST

Box GEP, Cox DR. (1964) An analysis of transformations. J Roy Stat Soc B Met 26: 211–252

Fournier DA, Skaug HJ, Ancheta J, Ianelli J, Magnusson A, Maunder MN, Nielsen A, Sibert J (2012) AD Model Builder: using automatic differentiation for statistical inference of highly parameterized complex nonlinear models, Optim Method Softw, 27: 233-249

Lokemoen JT, Koford RR (1996) Using candlers to determine the incubation stage of passerine eggs. J Field Ornithol 67:660-668

Polačiková L, Takasu F, Stokke BG, Moksnes A, Røskaft E, Cassey P, Hauber ME, Grim T (2013) Egg arrangement in avian clutches covaries with the rejection of foreign eggs. Anim Cogn 16: 819-828

Schneider CA, Rasband WS, Eliceiri KW (2012) NIH Image to ImageJ: 25 years of image analysis. Nat Methods 9: 671-675

Shapiro SS, Wilk MB (1965) An analysis of variance test for normality: Complete samples. Biometrika 52: 591-611

Skaug H, Fournier D, Nielsen A, Magnusson A (2011) Package glmmADMB: Generalized linear mixed models using AD Model Builder; software available at http://r-forge.r-project.org/projects/glmmadmb/

Stoddard MC, Stevens M (2010) Pattern mimicry of host eggs by the common cuckoo, as seen through a bird's eye. P Roy Soc London B Bio. 277: 1387-1393

Taylor CH, Gilbert F, Reader T (2013) Distance transform: a tool for the study of animal colour patterns. Methods Ecol Evol, 4: 771-781

Tseng Q, Wang I, Duchemin-Pelletier E, Azioune A, Carpi N, Gao J, Filhol O, Piel M, Théry M, Balland M (2011) A new micropatterning method of soft substrates reveals that different tumorigenic signals can promote or reduce cell contraction levels. Lab Chip 11: 2231-2240

Zhang D, Lu G (2002) Shape-based image retrieval using generic Fourier descriptor. Signal Process-Image 17: 825-848

Zitová B, Flusser J (2003) Image registration methods: a survey. Image Vision Comput, 21, 977-1000

Table 1

The relationship (Pearson's product-moment correlation) among the blunt pole distance, angle, orientation, and the adjacent angle. The upper right-hand portion represents the correlation coefficients and the bottom left-hand portion represents significances.

| Metric | Blunt pole distance | Blunt pole angle | Blunt pole orientation | Adjacent angle |
| --- | --- | --- | --- | --- |
| Blunt pole distance | NA | 0.28 | 0.36 | 0.13 |
| Blunt pole angle | < 0.0001 | NA | 0.56 | 0.26 |
| Blunt pole orientation | < 0.0001 | < 0.0001 | NA | 0.24 |
| Adjacent angle | 0.06 | 0.0001 | 0.0002 | NA |

Table 3

Generalized linear mixed model outputs predicting the behavioral response to experimental parasitism (either egg ejection or acceptance; top panel) or latency (egg ejections only, bottom panel), as the function of the absolute amount of disruption (continuous) rather than the treatment effects (categorical, Table 2), for the full model (left) and a reduced model (right) arrived at from a backward elimination process. For each mode we present the regression estimates, their approximate 95% family-wise confidence intervals, and their significances (bold values indicate significance that is 0.05 or less).

|  |  | Full model | | | |  | Final model | | | |
| --- | --- | --- | --- | --- | --- | --- | --- | --- | --- | --- |
|  |  |  |  |  |  |  |  |  |  |  |
|  |  | *Estimate* | *approximate CI 95%* | *χ^2^* | *P* |  | *Estimate* | *approximate CI 95%* | *χ^2^* | *P* |
| **Response**  Blunt pole distance | (Intercept) | 60453.02 | −15383.30 to 136289.34 |  | 0.12 |  | 7.04 | 0.003 to 14.072 |  | **0.05** |
|  | disruption | 0.01 | −0.47 to 0.49 | 0.00 | 0.96 |  | 0.04 | −0.44 to 0.52 | 0.02 | 0.88 |
|  | nesting age | −0.07 | −0.27 to 0.14 | 0.43 | 0.51 |  |  |  |  |  |
|  | laying date | −0.03 | −0.07 to 0.008 | 2.44 | 0.12 |  |  |  |  |  |
|  | clutch size | −1.78 | −3.31 to −0.25 | 5.18 | **0.02** |  | −1.71 | −3.23 to −0.18 | 4.82 | **0.03** |
|  |  |  |  |  |  |  |  |  |  |  |
| Blunt pole angle | (Intercept) | 60461.93 | −10215.67 to 131139.53 |  | 0.09 |  | 6.62 | −0.04 to 13.27 |  | **0.05** |
|  | disruption | 0.00 | −0.006 to 0.006 | 0.00 | 0.99 |  | < 0.0001 | −0.006 to 0.006 | 0.00 | 0.98 |
|  | nesting age | −0.07 | −0.26 to 0.12 | 0.57 | 0.45 |  |  |  |  |  |
|  | laying date | −0.03 | −0.07 to 0.005 | 2.81 | 0.09 |  |  |  |  |  |
|  | clutch size | −1.73 | −3.18 to −0.28 | 5.46 | **0.02** |  | −1.59 | −3.03 to −0.16 | 4.74 | **0.03** |
|  |  |  |  |  |  |  |  |  |  |  |
| Blunt pole orientation | (Intercept) | 60023.53 | −10628.59 to 130675.65 |  | 0.10 |  | 6.64 | 0.02 to 13.27 |  | **0.05** |
|  | disruption | 0.00 | −0.004 to 0.004 | 0.04 | 0.85 |  | 0.001 | −0.003 to 0.005 | 0.07 | 0.79 |
|  | nesting age | −0.07 | −0.26 to 0.12 | 0.56 | 0.45 |  |  |  |  |  |
|  | laying date | −0.03 | −0.07 to 0.005 | 2.77 | 0.10 |  |  |  |  |  |
|  | clutch size | −1.74 | −3.18 to −0.29 | 5.53 | **0.02** |  | −1.60 | −3.03 to −0.17 | 4.84 | **0.03** |
|  |  |  |  |  |  |  |  |  |  |  |
| Adjacent Angle | (Intercept) | 60513.70 | −10165.86 to 131193.26 |  | 0.09 |  | 6.61 | −0.05 to 13.26 |  | **0.05** |
|  | disruption | 0.00 | −0.006 to 0.006 | 0.002 | 0.96 |  | 0.000 | −0.006 to 0.006 | 0.00 | 0.98 |
|  | nesting age | −0.07 | −0.26 to 0.12 | 0.57 | 0.45 |  |  |  |  |  |
|  | laying date | −0.03 | −0.07 to 0.005 | 2.82 | 0.09 |  |  |  |  |  |
|  | clutch size | −1.73 | −3.18 to −0.28 | 5.48 | **0.02** |  | −1.59 | −3.02 to −0.16 | 4.75 | **0.03** |
|  |  |  |  |  |  |  |  |  |  |  |
|  |  |  |  |  |  |  |  |  |  |  |
| Dissimilarity | (Intercept) | 60023.53 | −10628.59 to 130675.65 |  | 0.10 |  | 6.64 | 0.02 to 13.27 |  | **0.05** |
|  | disruption | < 0.0001 | −0.004 to 0.004 | 0.04 | 0.85 |  | < 0.0001 | −0.003 to 0.005 | 0.07 | 0.79 |
|  | nesting age | −0.07 | −0.26 to 0.12 | 0.56 | 0.45 |  |  |  |  |  |
|  | laying date | −0.03 | −0.07 to 0.005 | 2.77 | 0.10 |  |  |  |  |  |
|  | clutch size | −1.74 | −3.18 to −0.29 | 5.53 | **0.02** |  | −1.60 | −3.03 to −0.17 | 4.84 | **0.03** |
| **Latency to ejection** | |  |  |  |  |  |  |  |  |  |
| Blunt pole distance | (Intercept) | 20604.75 | −8773729.87 to 8814939.37 |  | 1.00 |  | 14.97 | −50.77 to 80.70 |  | 0.66 |
|  | disruption | −0.53 | −44.29 to 43.24 | 0.001 | 0.98 |  | −0.31 | −43.84 to 43.22 | 0.0002 | 0.99 |
|  | nesting age | −0.16 | −23.94 to 23.63 | < 0.0001 | 0.99 |  |  |  |  |  |
|  | laying date | −0.01 | −4.38 to 4.36 | < 0.0001 | 1.00 |  |  |  |  |  |
|  | clutch size | 0.42 | −162.73 to 163.58 | < 0.0001 | 1.00 |  |  |  |  |  |
|  |  |  |  |  |  |  |  |  |  |  |
| Blunt pole angle | (Intercept) | 12637.81 | −8806403.40 to 8831679.02 |  | 1.00 |  | 15.01 | −49.12 to 79.13 |  | 0.65 |
|  | disruption | −0.01 | −0.46 to 0.45 | 0.001 | 0.98 |  | −0.01 | −0.47 to 0.46 | 0.0004 | 0.98 |
|  | nesting age | −0.15 | −23.87 to 23.56 | < 0.0001 | 0.99 |  |  |  |  |  |
|  | laying date | −0.01 | −4.388 to 4.37 | < 0.0001 | 1.00 |  |  |  |  |  |
|  | clutch size | 0.47 | −162.08 to 163.02 | < 0.0001 | 1.00 |  |  |  |  |  |
|  |  |  |  |  |  |  |  |  |  |  |
| Blunt pole orientation | (Intercept) | 17758.10 | −8815147.13 to 8850663.32 |  | 1.00 |  | 14.89 | −49.98 to 79.77 |  | 0.65 |
|  | disruption | 0.00 | −0.50 to 0.49 | < 0.0001 | 0.99 |  | −0.001 | −0.49 to 0.48 | < 0.0001 | 1.00 |
|  | nesting age | −0.16 | −24.14 to 23.82 | < 0.0001 | 0.99 |  |  |  |  |  |
|  | laying date | −0.01 | −4.40 to 4.38 | < 0.0001 | 1.00 |  |  |  |  |  |
|  | clutch size | 0.37 | −163.19 to 163.93 | < 0.0001 | 1.00 |  |  |  |  |  |
|  |  |  |  |  |  |  |  |  |  |  |
| Adjacent Angle | (Intercept) | 14863.14 | −8811310.66 to 8841036.95 |  | 1.00 |  | 14.82 | −49.27 to 78.91 |  | 0.65 |
|  | disruption | 0.00 | −0.56 to 0.56 | < 0.0001 | 1.00 |  | 0.002 | −0.57 to 0.57 | < 0.0001 | 1.00 |
|  | nesting age | −0.16 | −24.05 to 23.73 | < 0.0001 | 0.99 |  |  |  |  |  |
|  | laying date | −0.01 | −4.39 to 4.38 | < 0.0001 | 1.00 |  |  |  |  |  |
|  | clutch size | 0.29 | −163.93 to 164.50 | < 0.0001 | 1.00 |  |  |  |  |  |
|  |  |  |  |  |  |  |  |  |  |  |
|  |  |  |  |  |  |  |  |  |  |  |
| Dissimilarity | (Intercept) | 33258.75 | −18474.88 to 84992.37 |  | 0.21 |  | 5.17 | 1.23 to 9.12 |  | **0.01** |
|  | disruption | 0.13 | −3.34 to 3.60 | 0.005 | 0.94 |  | 1.21 | −2.01 to 4.43 | 0.53 | 0.46 |
|  | nesting age | −0.03 | −0.15 to 0.10 | 0.16 | 0.68 |  |  |  |  |  |
|  | laying date | −0.02 | −0.04 to 0.009 | 1.52 | 0.21 |  |  |  |  |  |
|  | clutch size | −1.18 | −2.10 to −0.26 | 6.93 | **0.01** |  | −1.18 | −2.07 to −0.29 | 7.02 | **0.01** |
