# Supplementary material for: Who moved my eggs? An experimental test of the egg arrangement hypothesis for the rejection of brood parasitic eggs
Source: Anim Cogn. 2014 Sep 7;18(1):299–305. doi: 10.1007/s10071-014-0800-x (PMC4674666; doi:10.1007/s10071-014-0800-x)
Supplement: Supplementary file 3 — ImageMagick codes used to calculate dissimilarity (for details see Methods). To use these codes you must first have ImageMagick installed, and then extract this zipped file. These are bash codes intended to be run on a Linux operating system, but these can be customized for other operating systems. (ZIP 13 kb) [file 10071_2014_800_MOESM3_ESM.zip › 10071_2014_800_MOESM3_ESM/EggArrangev0.1/README.rtf]

Electronic Supplementary Material 3

Who moved my eggs? An experimental test of the clutch arrangement hypothesis for the rejection of brood parasitic eggs


Daniel Hanley (1), Peter Samaš (1), Mark E. Hauber (2), Tomáš Grim (1)

Affiliations and addresses:
 (1) Department of Zoology and Laboratory of Ornithology, Palacký University, 17. listopadu 50, Olomouc, 77146, Czech Republic

 (2) Department of Psychology, Hunter College and the Graduate Center, The City University of New York, 695 Park Avenue, New York, New York 10065, United States


Email address: danielhanley00@gmail.com

Desription: These functions (together called, EggArrange version 0.1) run a modified distance transformation (Taylor et al. 2013). They were written use ImageMagick (http://www.imagemagick.org/), and are written for Ubuntu (12.04 -64 bit, Precise Pangolin). Some modifications of these codes may be necessary for your local system and operating system. Please contact us if you need help using EggArrange version 0.1.
Usage: Currently, EggArrange requires user input to specify the name of the nest that a user wishes to compare. And these images must be placed in the “input” folder. Pairs of images (e.g., before and after) must have similar names and differ only by the number 1 or 2 before the extension (tif). Currently, the default name must be [NEST NUMBER].tifarrangement[1 or 2].tif, which can be created by our ImageJ functions (Electronic Supplementary Materials 2). EggArrange can be used in a loop to process all the files (with a common naming structure) in a folder.
Reference:
Taylor CH, Gilbert F, Reader T (2013) Distance transform: a tool for the study of animal colour patterns. Methods Ecol Evol, 4: 771-781
